# Clinical expert consensus document on quantitative coronary angiography from the Japanese Association of Cardiovascular Intervention and Therapeutics

**DOI:** 10.1007/s12928-020-00653-7

**Published:** 2020-03-03

**Authors:** Nobuaki Suzuki, Taku Asano, Gaku Nakazawa, Jiro Aoki, Kengo Tanabe, Kiyoshi Hibi, Yuji Ikari, Ken Kozuma

**Affiliations:** 1 Department of Fourth Internal Medicine, Teikyo University Mizonokuchi Hospital, Kawasaki, Japan; 2 Department of Cardiology, St. Luke’s Hospital, Tokyo, Japan; 3 Department of Cardiovascular Medicine, Kindai University Faculty of Medicine, Osaka-Sayama, Japan; 4 Division of Cardiology, Mitsui Memorial Hospital, Tokyo, Japan; 5 Division of Cardiology, Yokohama City University Medical Center, Yokohama, Japan; 6 Department of Cardiology, Tokai University Hospital, Isehara, Japan; 7 Division of Cardiology, Department of Medicine, Teikyo University School of Medicine, 2-11-1, Kaga, Itabashi-ku, Tokyo, Japan

**Keywords:** Quantitative coronary angiography, Coronary artery disease, Coronary artery stents, Expert consensus document

## Abstract

Quantitative coronary angiography (QCA) remains to play an important role in clinical trials and post-marketing surveillance related to the safety and efficacy of new PCI devices. In this document, the current standard methodology of QCA is summarized. In addition, its history, recent development and future perspectives are also reviewed.

## Introduction

Percutaneous coronary intervention (PCI) is the standard of treatment for ischemic heart disease. An accurate assessment of stenosis and the reference diameter is important; hence, evaluation of coronary angiography is essential for PCI. In clinical settings, quantitative coronary angiography (QCA) is typically used to assess coronary artery stenosis. The visual assessment of lumen diameter and stenosis is not objective. Therefore, QCA was developed for the objective evaluation of lumen diameter. Truly, a report of the European Society of Cardiology–European Association of Percutaneous Cardiovascular Interventions Task Force on the Evaluation of Coronary Stents in Europe supports the importance of independent QCA analysis as follows; “Offline quantitative coronary analysis in a centralized core laboratory with blinded outcome assessors in case of comparative studies is mandatory” [1, 2]. Also, the US Food and Drug Administration addresses that the independent quantitative angiographic assessment is momentous [2, 3]. Accordingly, the Academic Research Consortium-2 Consensus Document recommends the use of independent core laboratory-verified QCA analysis using the hierarchical approach when the trial protocols are not incorporate the mandatory use of fractional flow reserve (FFR), or any other functional assessment [2]. In short, the hierarchical approach of the definition with respect to clinically indicated repeat revascularization is following: (1) QCA [preferably three-dimensional (3D) QCA] diameter stenosis > 50% (based on the average of multiple views) with either recurrent symptoms or positive noninvasive functional test; (2) QCA (preferably 3D QCA) diameter stenosis > 70% (based on the average of multiple views) regardless of other criteria; (3) QCA diameter stenosis > 70% (based on the worst view) regardless of other criteria. Thus, QCA remains to play an important role in clinical trials and post-marketing surveillance related to the safety and efficacy of new PCI devices.

## History and future perspectives

Selective coronary angiography was born in 1959 [4], and generally accepted as the gold standard for the assessment of coronary artery disease. The American Heart Association reporting system for grading coronary artery disease was established in 1975 to standardize the evaluation of coronary angiography [5]. Of note, the visual assessment of coronary angiography is not necessarily strictly objective and intra- and inter-observer variabilities have become a great concern. Zie et al. reported that all observers agree regarding the significance of stenosis (defined as > 50% narrowing of the luminal diameter) in the proximal or mid-left anterior descending coronary artery in only 65% of coronary angiograms. Moreover, there was disagreement by at least one observer concerning the significance of lesions noted in the main left coronary artery in 15% of angiograms [6]. In addition, the introduction of balloon angioplasty and advances in medical therapy for atherosclerosis, offering potential for plaque regression, also required an objective and reproducible approach to accurately describe the dimensions of coronary arteries. In 1971, Gensini et al. reported an electronic caliper system in which the arterial border and normal segment of the lesion were manually defined by moving the cursor [7]. The first validation study of QCA using digital computation was performed in 1977 [8]. In the 1980s, the Digital Imaging and Communications in Medicine (DICOM) system and novel contour detection algorithm [9] were developed to measure vessels with complex contour (e.g., post balloon dilatation, ruptured plaque), which are usually observed in daily clinical practice. Kondo et al. reported that it is reasonable to allow the edge-detection algorithm determine the measurements in types B and C dissected lesions in terms of predicting long-term patency after angioplasty [10]. These new technologies have contributed to the dramatic progress achieved in QCA.

In the 1990s, new devices [e.g., metallic stents, drug-eluting stents (DES)] have been established to eliminate restenosis after PCI [11, 12]. QCA contributed greatly to the development of new devices and techniques by accurately measuring the late lumen loss and diameter of stenosis and judging those efficacies [13–33]. In the 2000s, it was established that evaluation of ischemia using FFR is useful for selecting appropriate lesions for PCI [34, 35]. Subsequently, 3D QCA, which was first conceived earlier [36], was applied to clinical practice. In the 2010s, the angiography-derived FFR method based on 3D QCA was introduced for a more accurate assessment of clinically significant stenosis [37]. Tu et al. showed that the angiography-derived FFR exhibits good correlation with FFR [38].

**Fig. 1:**
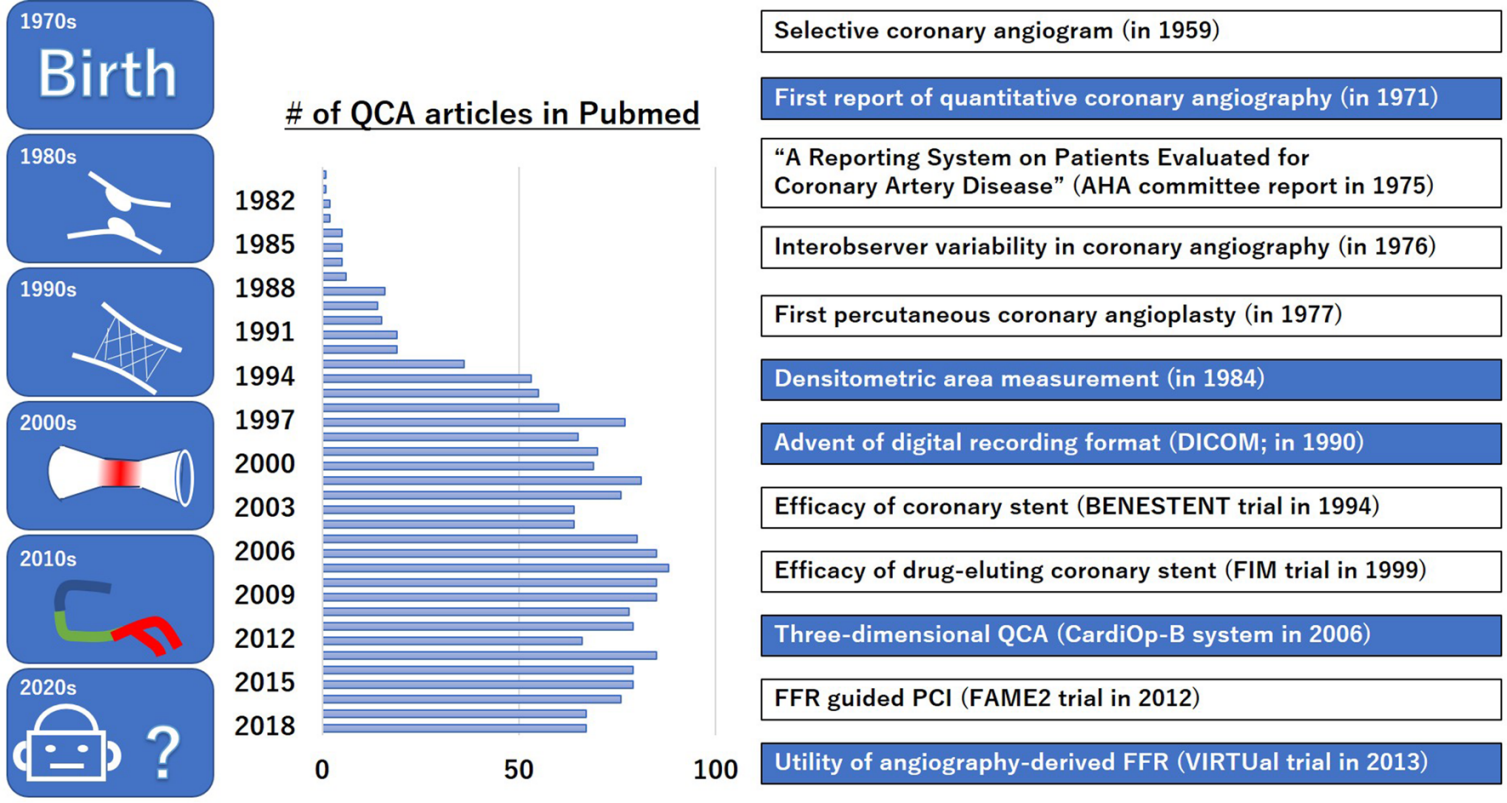
History of quantitative coronary angiography. Quantitative coronary angiography (QCA) originated from the basic concept of showing objective numerical data in addition to a visual assessment. The emergence of metallic stents and drug-eluting stents has further increased its utility. As shown by the number of articles available in PubMed, even after the introduction of intravascular imaging and fractional flow reserve, QCA has maintained its value by applying three-dimensional QCA technology. *FFR* fractional flow reserve, *PCI* percutaneous coronary intervention

Figure 1 shows the quick history of QCA. Future developments in QCA in the 2020s remain unknown. QCA may offer the potential for further development, through the application of artificial intelligence and the Internet, to improve the accuracy of decision-making in PCI. Advances in image enhancement continue to improve the usefulness of online quantification, while there is still room for development. Under the promotion of “Society 5.0” in Japan, online digital systems and computer application packages may become widely available in the future through commercial distribution. In combination with artificial intelligence, these systems may be designed to optimize the PCI procedure.

### Calibration

Calibration is necessary to obtain measurement data through QCA analysis. Nowadays, progress in the isocenter technique method enables automatic image calibration by applying the data available in the DICOM file. Isocenter calibration transforms cardiac structures in digitized biplane angiograms into absolute dimensions, calculating their radiological magnification and video transformation [39]. Conventionally, this technique is based on two fixed reference points in the center of the two image intensifiers [40]. Application of flat panel and contemporary technologies ensured the accuracy of analysis through isocenter calibration, and led to the development of 3D QCA [41]. Accordingly, if the isocenter calibration is applicable, the catheter size does not need to be considered. Only when the isocenter calibration is applied, the size per pixel (calibration factor) in the cine must be evaluated using an object of predetermined size. These objects need to be visualized on the same picture with the target vessel. Calibration using a catheter was the most common approach in past era. Thus, it is necessary to capture images with a straightly positioned catheter in the Valsalva sinus. The recommended calibration factor for standard analysis software is 0.20 ± 0.02 mm/pixel. In such situation, the analyst needs to enter the size of the catheter in the image into the program. The size of the catheter is preferably ≥ 6 French. Indeed, Ito et al. reported that 4 French catheters are less reliable than 6 French catheters when measuring QCA data especially for follow-up data that need most accurate measurements of minimal lumen diameter and diameter stenosis [42]. Additional information regarding the catheter is not required, although a previous report showed the influence of the catheter type on the calibration [43].

### Analytical process and measurement data

Initially, the edge of the contrasted blood vessel is drawn using an automatic edge-detection algorithm. Following the determination of a start point and an end point in the image of enhanced coronary artery, a vessel pathline is created. Subsequently, the vessel contour is delineated in accordance with the pathline. The pathline and vessel contour, which are determined automatically in accordance with the contrast density, occasionally requires editing by analysts. Lumen and reference diameters are displayed in the graph, which is helpful for editing. The automatically determined lesion length may also require review and editing by analysts. Especially at the post-PCI or follow-up phases, the border of the device must be manually pointed by the analysts, and the proximal and distal edge subsegments are subsequently determined. Generally, the following parameters are obtained through QCA: (1) minimal lumen diameter (the smallest diameter of the lumen); (2) reference diameter (the average diameter of the lumen assumed without atherosclerotic disease); (3) obstruction length (the QCA software automatically recognizes the two borders between the normal and diseased vessel by detecting the directional change in the coronary artery contour, and measures the length of stenosis). The following formulas provide the parameters enhancing the severity of obstruction and acute/chronic outcome of interventional therapeutics: (1) diameter stenosis = (reference diameter − minimal lumen diameter)/reference diameter; (2) acute gain = post-PCI minimal lumen diameter − baseline minimal lumen diameter; (3) late loss = post-PCI minimal lumen diameter − follow-up minimal lumen diameter. A more detailed report of some software also shows the mean lumen diameter.

**Fig. 2:**
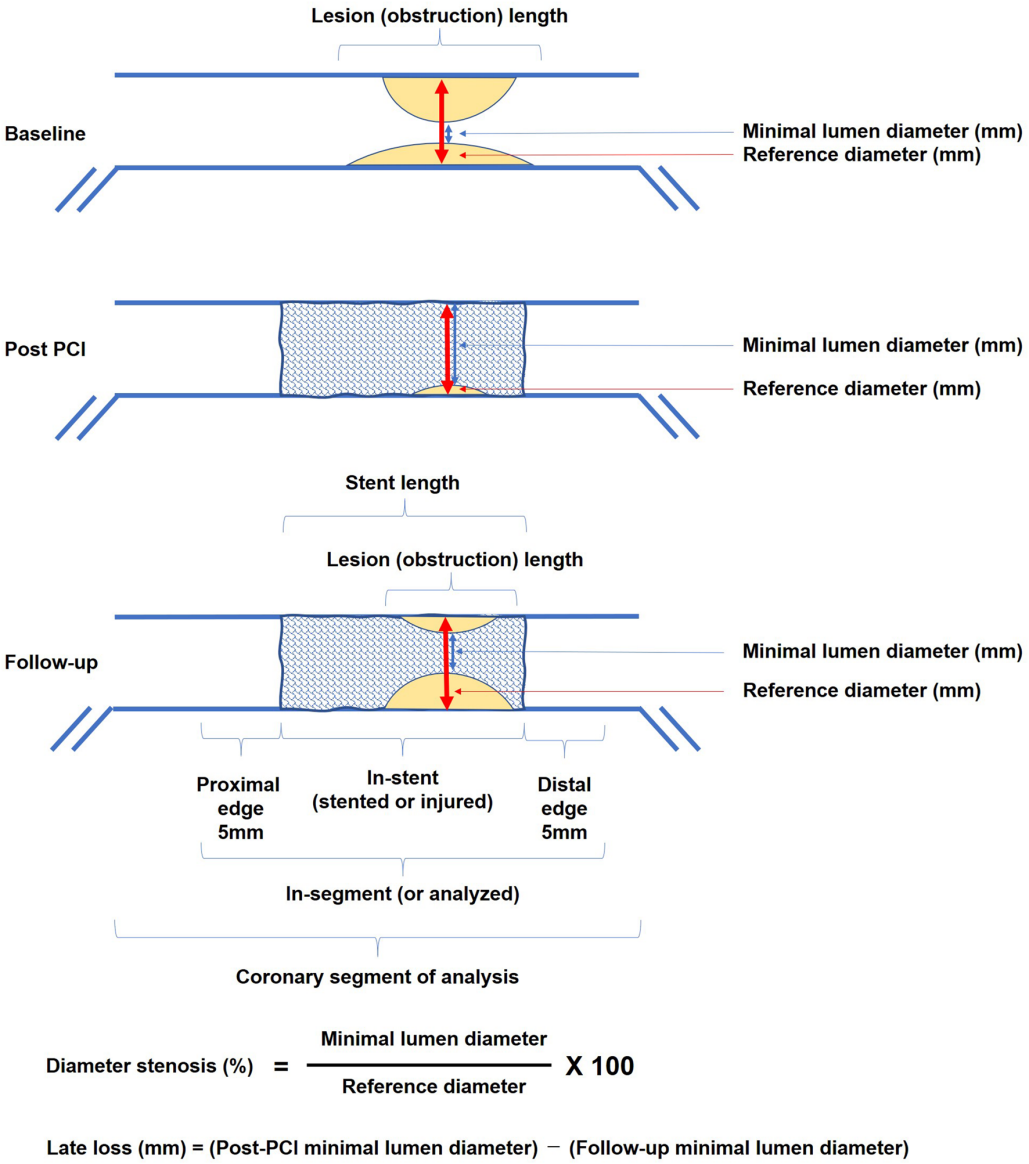
Standard scheme of measurement data. *PCI* percutaneous coronary intervention

A standard scheme is shown in Fig. 2. Following the emergence of brachytherapy and DES technologies, restenosis appeared at edge subsegment was more focused on [44, 45]. In the current standard system using the DES analysis algorithm, a stent edge of 5 mm on both ends is automatically selected after the selection of the stent range. Conventionally, the analysis area was subjectively set by the analyst using the nearby side branches as landmarks. However, this algorithm automatically defines the analysis range of the stent and its surroundings, enabling more objective comparison.

Accurate measurement of the minimal lumen diameter and reference diameter leads to the accurate evaluation of late lumen loss and diameter of the stenosis, which are accepted by the medical community as efficacy endpoints. Late loss has been associated with long-term clinical events [46]. In fact, QCA made a great contribution in comparing the utility of each type of DES inhibiting neointimal hyperplasia in the early 2000s. Thus, contemporary clinical trials have shown the effectiveness of the new coronary device compared with that of devices of the previous generation based on the concept of late loss.

### Tips for obtaining optimal QCA data

Operators must perform high-quality coronary angiography for accurate QCA. The images without guide wire need to be selected. Notably, use of an insufficient amount of contrast medium usually results in incorrect determination of the vessel contour, which requires extensive editing. The setting of the auto injector may have to be altered in advance, especially for large vessels or patients with arteriovenous shunts undergoing hemodialysis. Crossing the side branch, which can result in an incorrect direction of the pathline, should be avoided. The angiographic conditions need to be consistent at the post-PCI and follow-up phases to accurately determine the late loss. Therefore, the operator must ensure the consistency of the oblique used for QCA in each phase.

In clinical practice, QCA data can be influenced by the frame selection [47]. In the selection of the target frame, the analyst should (1) minimize vessel movement, (2) select the contrast-filled vessels in the end-diastolic phase, and (3) appropriately include the calibration device when the isocenter calibration is not applicable.

One may think that differences in software programs influence the QCA measurements. However, Kozuma et al. reported that measurements of the minimal lumen diameter and reference diameter did not show major systematic differences between currently available software programs [48].

QCA measurements may not necessarily be consistent with those of other modalities. Kubo et al. reported that the measurement values in QCA are lower compared with those obtained through optical coherence tomography and intravascular ultrasound [49]. Furthermore, Sotomi et al. reported that the discrepancy between QCA and optical coherence tomography in terms of lumen loss was minor; however, it tended to be greater in the analysis of lesions with a bioresorbable scaffold [50].

**Fig. 3:**
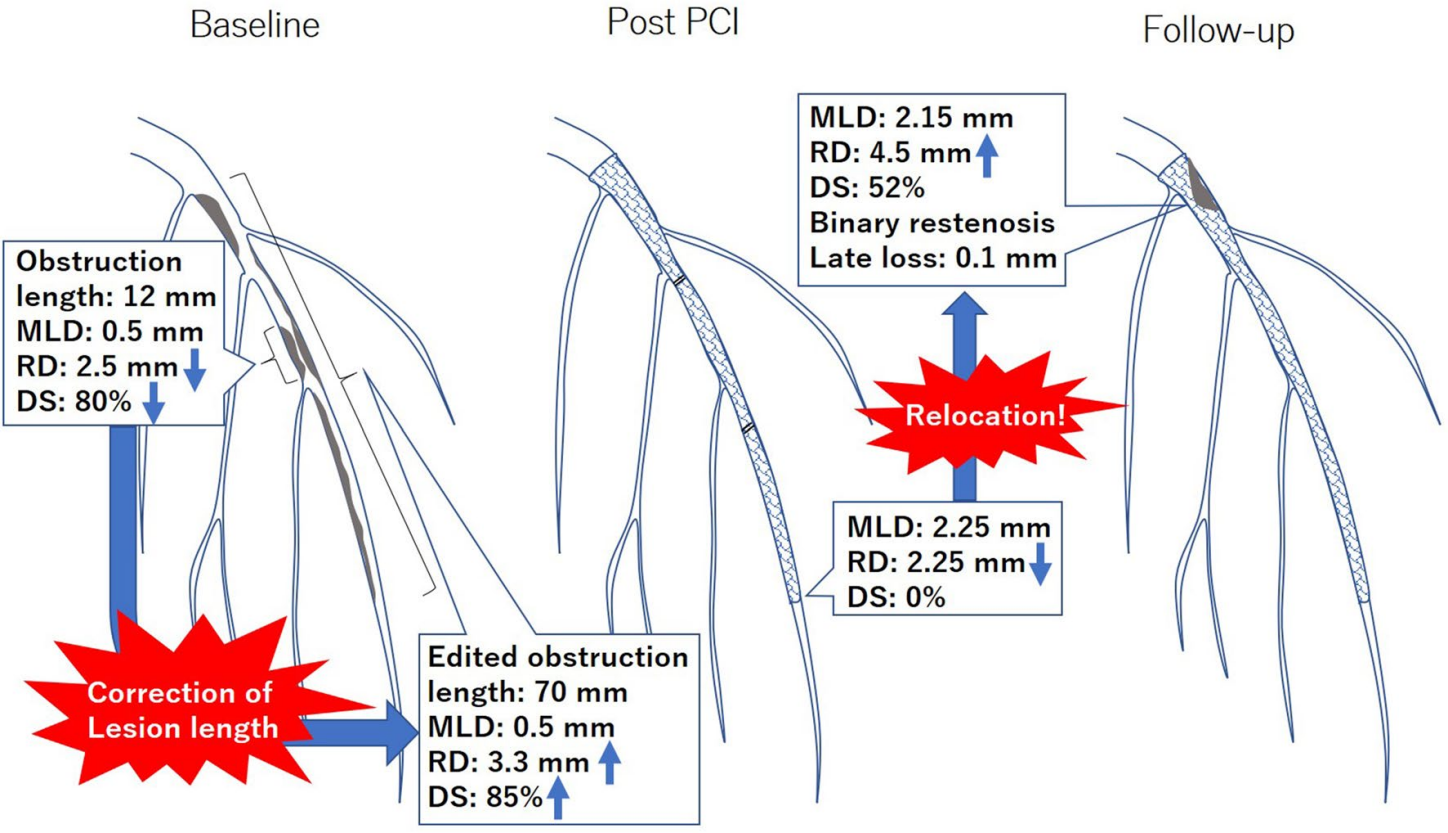
A representative case of diffuse lesion. When the automated obstruction length and reference diameter are too underestimated at baseline, appropriate correction of the lesion length should be considered. When the in-stent restenosis appears proximally on diffuse lesion, the value of late lumen loss is usually unremarkable, whereas the diameter of stenosis is prominent. This discrepancy is so-called the previously reported “relocation” of the minimal lumen diameter. *MLD* minimal lumen diameter, *RD* reference diameter, *DS* diameter stenosis

Currently, there are several concerns regarding the interpretation of QCA measurements for diffuse lesions (Fig. 3). At baseline, the automated obstruction length and reference diameter are occasionally underestimated. Therefore, appropriate correction of the lesion length should be considered. Discrepancy may be observed in the diameter of stenosis at the follow-up phase and late loss. In case of a proximal restenosis after the stent implantation on diffuse lesion, the value of late lumen loss is usually unremarkable, whereas the diameter of stenosis is prominent. Concretely, the in-stent minimal lumen diameter is usually located distally at the post-PCI phase due to vessel tapering. However, when the restenosis appears far proximally, the value of late loss tends to be low despite binary restenosis. This discrepancy is so-called the previously reported “relocation” of the minimal lumen diameter [51]. Such discrepancy is usually more exaggerated in the measurement of the in-segment, when the minimal lumen diameter exhibits a markedly low value at the distal edge subsegment post-PCI.

**Fig. 4:**
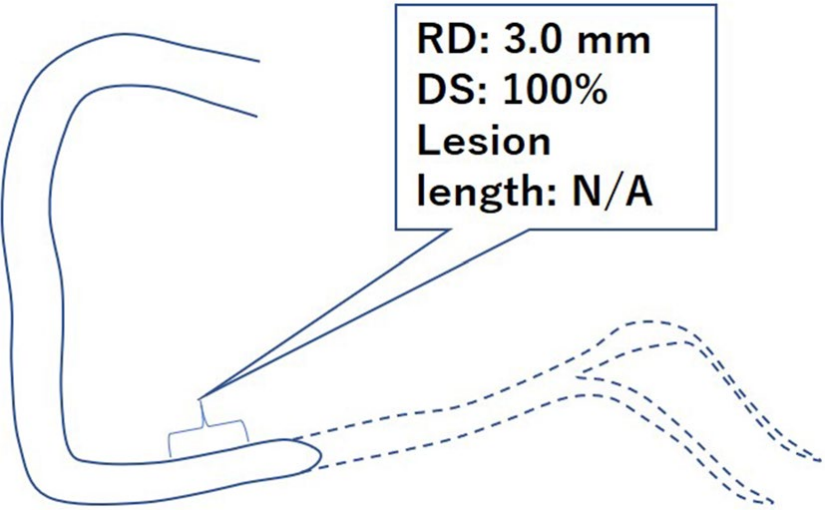
An example of total occlusive lesion. *RD* reference diameter, *DS* diameter stenosis

Distal reference may not be necessarily included in the analysis of total occlusion. In such a case, the reference segment where the vessel size is supposed to be consistent with the occluded segment needs to be selected and only the reference diameter is to be measured. Subsequently, the diameter of the stenosis and minimal lumen diameter are manually determined as 100% and 0 mm, respectively (Fig. 4).

The followings should be considered to optimize the results of QCA:

- To facilitate the frame selection, the frame rate should be ≥ 12.5/s.
- The flat panel should be positioned to be the closest to patient.
- When the isocenter calibration is not applicable, the catheter size is preferably ≥ 6 French.
- The setting of the auto-injector should be altered if the target vessel requires a large amount of contrast agent (e.g., large vessels, patients with arteriovenous shunts).
- Adequate oblique should be selected to avoid other branch crossing or foreshortening of the target lesion.
- In advance, actual stent length and diameter of devices should be reviewed.
- The position of the flat panel and oblique and any other conditions should be consistent between baseline, post-PCI, and follow-up angiography. Inconsistency is occasionally responsible for unreasonable data.

### Bifurcation analysis

The coronary tree is characterized by its natural fractal geometric pattern. In the coronary tree, whole upstream blood flow is distributed to downstream bifurcated branches. It is established that, in bifurcated vessel, the sizes of the daughter vessels (i.e., distal main branch and side branch) are smaller than that of the mother vessel (i.e., proximal main branch). Huo and Kassab et al. reported the relationship in diameter (*D*) between the mother and daughter vessels as described below [52]:

$$D\,{\text{mother}}\,{\text{vessel}}^{{\frac{7}{3}}} = D\,{\text{larger}}\,{\text{daughter}}\,{\text{vessel}}^{{\frac{7}{3}}} + D\,{\text{smaller}}\,{\text{daughter}}\,{\text{vessel}}^{{\frac{7}{3}}} .$$

According to this principle, the coronary artery changes its diameter at the point of bifurcation (step-down phenomenon). However, conventional single-vessel QCA algorithms were developed based on the assumption of minimum vessel tapering. It was reported that the accuracy of the single-vessel QCA algorithms in bifurcated lesion was limited [53]. Ishibashi et al. reported that the single-vessel QCA algorithms underestimated the diameter of stenosis in the proximal mother vessel due to underestimated interpolated reference vessel diameter and overestimated diameter stenosis in the distal daughter vessels due to overestimated reference vessel diameter [54]. Another limitation of the single-vessel QCA algorithms is the requirement of a non-existing vessel contour crossing a bifurcation core. This results in the need for manual corrections, potentially introducing bias [54].

**Fig. 5:**
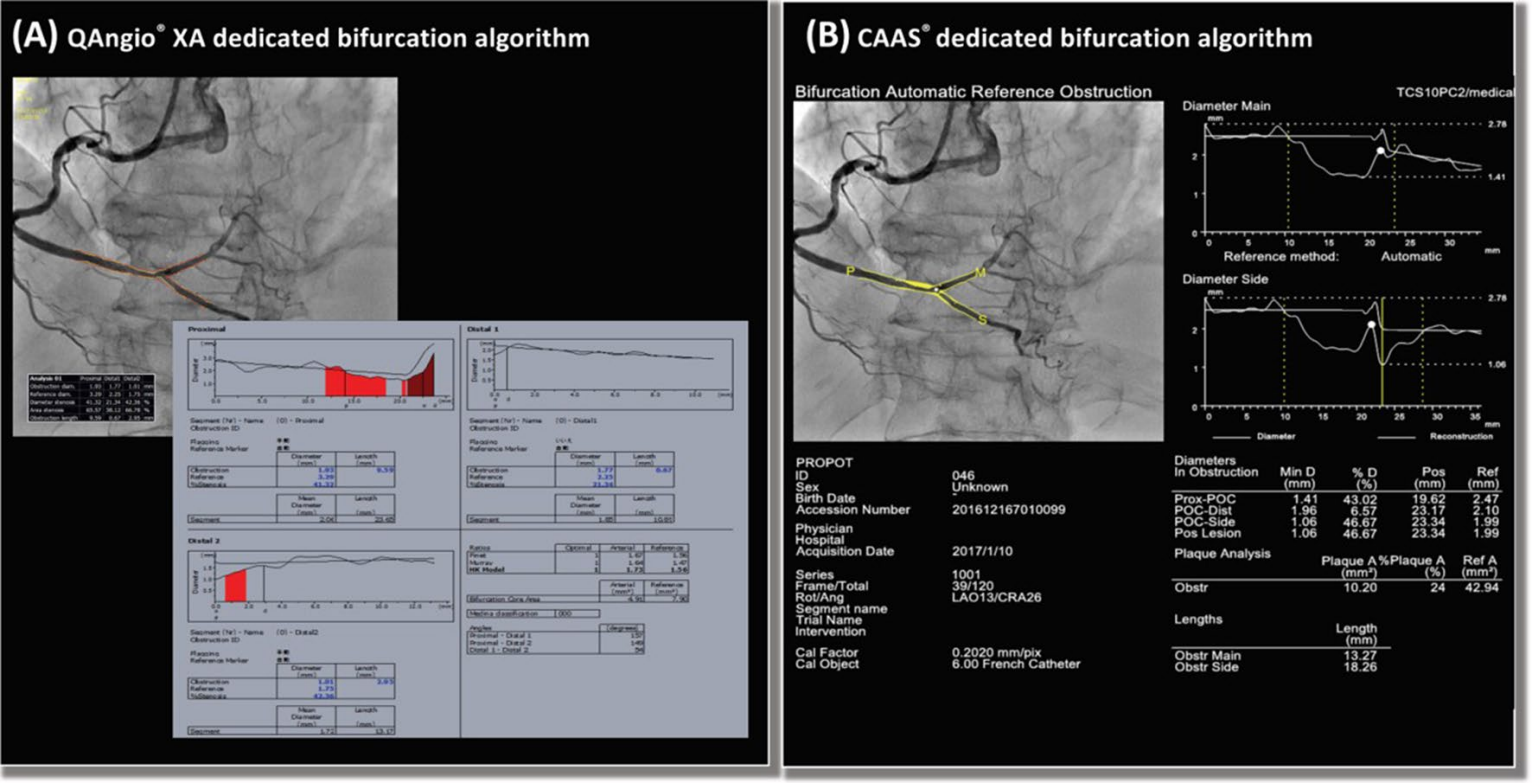
Bifurcation analyses using bifurcation-dedicated algorithms on **a** QAngio^®^ XA and **b** CAAS^®^

Dedicated bifurcation QCA algorithms were developed to overcome these limitations. Major QCA software, such as QAngio^®^ XA (Medis Medical Imaging systems B.V, Leiden, the Netherlands) and CAAS^®^ (Pie Medical Imaging B.V, Maastricht, the Netherlands) optionally incorporate a two-dimensional (2D) bifurcation analysis package (Fig. 5). These dedicated bifurcation algorithms are based on a common concept that stenoses are individually assessed in each segment (i.e., proximal major branch, distal major branch, and side branch), with the segment-specified interpolated reference vessel diameter and vessel contours delineating actual bifurcation geometry avoiding the non-existing vessel contours across a bifurcation core. These algorithms have been validated using precision-manufactured bifurcation phantoms and compared with conventional single-vessel QCA algorithms [54]. In this validation study, the dedicated bifurcation algorithms yielded superior accuracy and precision than single-vessel QCA algorithms. Although the two different algorithms (i.e., QAngio^®^ XA and CAAS^®^) feature their original methodologies, they exhibited comparable analytic performance in the validation study [54].

**Fig. 6:**
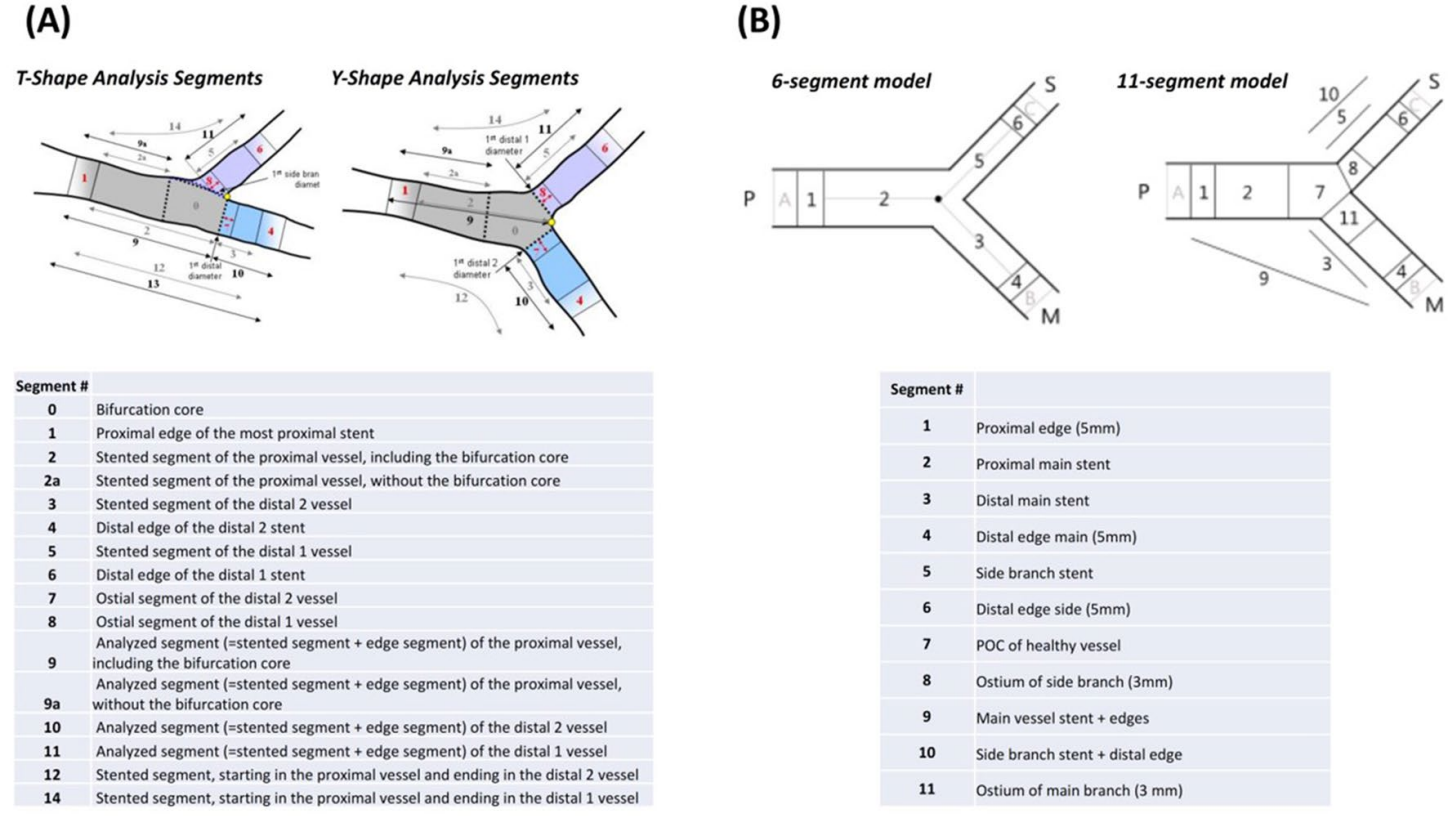
Subsegments defined using bifurcation-dedicated algorithms on **a** QAngio^®^ XA and **b** CAAS^®^. **a** QAngio^®^ XA provides two different bifurcation analysis models (T-shape model and Y-shape model) that are applied according to the morphology of the bifurcation. **b** CAAS^®^ reports two different segment models (6- and 11-segment model). *POC* polygon of confluence
- For the serial quantitative assessment of the efficacy of the intervention, the analyses should be performed at the time of pre-intervention, post-intervention, and follow-up with the same projection angle.

For the appropriate quantitative angiographic assessment of bifurcated vessels, the consensus group recommends the following:

- The bifurcation should be assessed in the optimal angiographic view orthogonal to the bifurcation plane in which the widest bifurcation angle is visualized without overlapping or foreshortening of vessels.
- The dedicated bifurcation algorithm rather than the conventional single-vessel algorithm should be used, especially in the case of major bifurcation (e.g., left main coronary artery and major bifurcation in the proximal left anterior descending and left circumflex arteries).
- The quantitative and qualitative (i.e., calcification, thrombus dissection, etc.) results should be independently reported in each of three segments (i.e., proximal main branch, distal main branch, side branch), and the Medina classification (based on the quantitative analysis) should be reported [55]. For the additional report, detailed subsegments of each branch are defined using each of the software packages (14 subsegments on QAngio^®^ XA and 6 or 11 subsegments on CAAS^®^) (Fig. 6).

### Aorto-ostial analysis

The QCA in aorto-ostial lesions requires several considerations. The ostial lesion should be assessed with the optimal viewing angle perpendicular to the ostium of the coronary artery. For the ostium of the left main coronary artery, AP, cranial 35°–45° and LAO 30°–45°, cranial 25°–35° are options for the optimal view. [56] For the quantitative analysis, it is often difficult to define a proximal reference vessel due to an insufficient length of the healthy vessel proximal to the obstruction. This precludes researchers from defining an appropriately interpreted reference vessel diameter. When the automatic interpolated reference line is inappropriate, manual corrections of the reference line are needed. In the CAAS^®^, the “local reference” function enables to modify the reference diameter by selecting a reference diameter location that the operator considers as a healthy diameter. In the QAngio^®^ XA, the “flagging” function is used for the correction of the reference diameter. Furthermore, the QAngio^®^ XA optionally incorporates the dedicated algorithm for ostial analysis. According to this algorithm, the reference diameter is interpolated based on the proximal segment with a limited length and distal reference segment.

## 3D quantitative coronary angiography

The 3D QCA was developed to resolve several drawbacks of 2D QCA. In 3D QCA, at least two angiographic projections are required to reconstruct a 3D geometry [57, 58]. The 3D geometry allows an accurate quantitative assessment of the eccentric cross-sectional luminal morphology of the coronary artery, which hinders precise analysis in 2D QCA. Furthermore, it averts inaccurate length measurement due to vessel shortening, which is frequently observed in 2D QCA. It was reported that the 3D QCA parameters more precisely reflected the luminal dimensions measured through intravascular ultrasound and FFR compared with 2D QCA [37, 59].

The advantage of 3D QCA is highlighted in the assessment of bifurcation. This is because the 3D reconstruction theoretically yields a precise geometric assessment, such as measurement of the bifurcation angle, which is commonly difficult in 2D QCA due to the overlap and foreshortening of branches. The superiority of the analytic performance of the 3D bifurcation algorithm over the 2D bifurcation algorithm in the assessment of bifurcation has been previously reported [60, 61]. Furthermore, the 3D reconstruction aids the angiographer and interventionist to determine the optimal projection angle.

### Angiography-derived FFR

In the 1980s and 1990s, the physiological assessment of coronary arteries was performed using “semi-quantitative” angiographic methodologies, such as Thrombolysis in Myocardial Infarction (TIMI) flow grade, TIMI frame count, and TIMI myocardial perfusion grade [62–64]. However, these methodologies are somewhat fuzzy, and thus have major limitations in providing accurate interpretation of the complex physiological phenomena [65, 66]. In 1993, N. Pijls and B. Bruyne invented FFR to assess the severity of physiological stenosis using a pressure wire [67, 68]. This index was widely accepted for its feasibility with objective values, low dependence on the skills of the operator, and robust relationship with clinical prognosis [69, 70]. Furthermore, other feasible and accurate wire-based indices using coronary thermodilution (i.e., coronary flow reserve and intracoronary microvascular resistance) were invented in the 2000s. These indices assisted in overcoming the limitations of the classic invasive coronary flow assessment with a Doppler wire. Subsequently, these novel wire-based indices replaced the classic angiographic physiological methodologies.

Following the advent of FFR, researchers attempted its computation based only on angiographic information (i.e., angiography-derived FFR). The initial angiography-derived FFR was developed based on the computer fluid dynamics with a complex mathematical formula (i.e., Navier–Stokes equation) which prolonged the calculation time to several days [71]. To overcome this limitation, Papafaklis et al. and Tu et al. reported the angiography-derived physiological indices with a simple mathematical formula based on Poiseuille’s and Bernoulli’s laws [38, 72]. These computed physiological indices, which could be acquired within 5–10 min, exhibited a satisfactory diagnostic performance in the validation studies, with the wire-based FFR used as Refs. [38, 72]. In the meta-analysis including angiography-derived FFR with various methodologies, the pooled sensitivity, specificity, and summarized area under the receiver-operating curve against the wire-based FFR were 0.89, 0.90, and 0.84, respectively [73]. In the meta-analysis, the angiography-derived FFR based on the simple equation demonstrated a comparable diagnostic performance with that of angiography-derived FFR based on the Navier–Stokes equation.

**Fig. 7:**
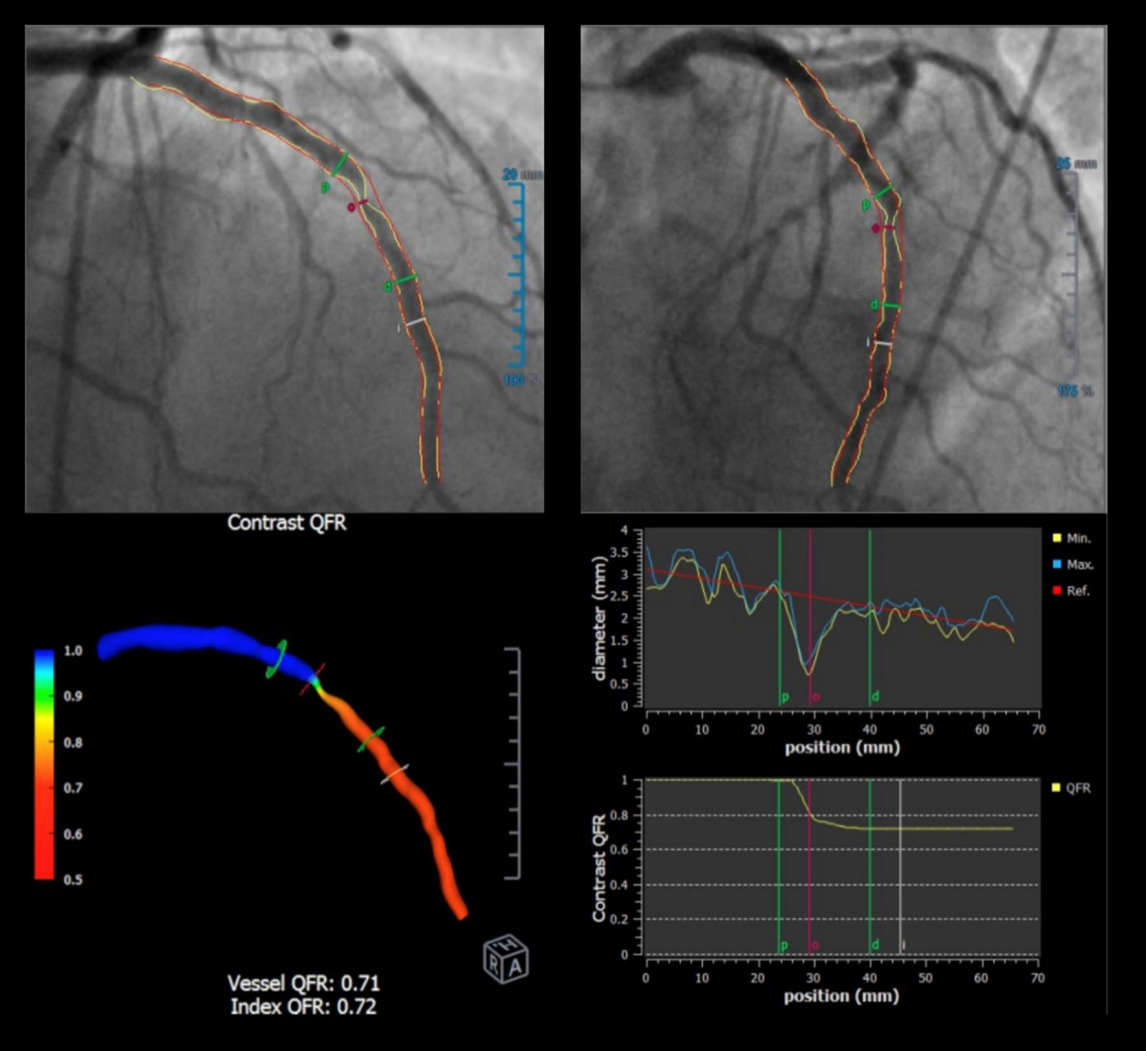
Quantitative flow ratio on QAngio^®^ XA. The quantitative flow ratio (QFR) is computed based on three-dimensional angiography reconstructed from two different projections with angles ≥ 25° apart and volumetric flow rate calculated using a contrast bolus frame count. The QFR reports virtual color-coded pullbacks of FFR of the arteries with stenosis for which angiography were performed, without the use of a pressure wire or hyperemia

Commercially available software packages for the angiography-derived FFR have been developed by Medis Medical Imaging System B.V. (QFR^®^: Quantitative Flow Ratio), Pie Medical Imaging B.V. (vFFR^®^), and CathWorks (FFRangio^®^) (Fig. 7). These software packages provide an online analysis system enabling the acquisition of a computed FFR value in the Cath lab. They are expected to support physicians or operators in decision-making for the indication of revascularization without using a pressure wire. Considering the advantages of less invasiveness and time-effectiveness, angiography-derived FFR potentially plays a role in specific situations, such as a non-culprit lesion assessment in the setting of acute coronary syndrome, post-PCI lesion assessment, and patient risk assessment using the functional SYNTAX score [74–78].

For a surrogate marker of stent efficacy, the potential applicability of the angiography-derived FFR as a new-generation QCA was reported [79]. A patient-level meta-analysis reported that even a statistically significant difference in angiographic late lumen loss within the low value range did not have a clinical impact [80]. Taking into account the non-linear relationship between luminal loss and deterioration of coronary flow, it may be worth incorporating a physiological assessment (e.g., angiography-derived FFR) into the quantitative assessment of efficacy in the current DES era [81].

Another advantage of angiography-derived FFR is that it can be also analyzed in a post hoc manner. Based on this advantage, angiography-derived FFR is a one of the recommended tools in the second version of the Academic Research Consortium consensus document on clinical endpoints in coronary intervention trials for the confirmation of “clinically-indicated” or “ischemia-driven” repeat revascularization in the adjudication of clinical events [2].
